# Predicting Undesired Treatment Outcomes With Machine Learning in Mental Health Care: Multisite Study

**DOI:** 10.2196/44322

**Published:** 2023-08-23

**Authors:** Kasper Van Mens, Joran Lokkerbol, Ben Wijnen, Richard Janssen, Robert de Lange, Bea Tiemens

**Affiliations:** 1 Behavioural Science Institute, Radboud University, Nijmegen, Netherlands; 2 Data Science, Altrecht Mental Healthcare, Utrecht, Netherlands; 3 Centre of Economic Evaluation & Machine Learning, Trimbos Institute (Netherlands Institute of Mental Health), Utrecht, Netherlands; 4 Department of Clinical Epidemiology and Medical Technology Assessment, Maastricht University Medical Centre, Maastricht, Netherlands; 5 Health Care Governance, Erasmus School of Health Policy and Management, Erasmus University Rotterdam, Rotterdam, Netherlands; 6 Scientific Centre for Care and Welfare, Tilburg University, Tranzo, Tilburg, Netherlands; 7 Alan Turing Institute, Almere, Netherlands; 8 Indigo Service Organization, Utrecht, Netherlands; 9 Pro Persona Research, Renkum, Netherlands

**Keywords:** treatment outcomes, mental health, machine learning, treatment, model, Netherlands, data, risk, risk signaling, technology, clinical practice, model performance

## Abstract

**Background:**

Predicting which treatment will work for which patient in mental health care remains a challenge.

**Objective:**

The aim of this multisite study was 2-fold: (1) to predict patients’ response to treatment in Dutch basic mental health care using commonly available data from routine care and (2) to compare the performance of these machine learning models across three different mental health care organizations in the Netherlands by using clinically interpretable models.

**Methods:**

Using anonymized data sets from three different mental health care organizations in the Netherlands (n=6452), we applied a least absolute shrinkage and selection operator regression 3 times to predict the treatment outcome. The algorithms were internally validated with cross-validation within each site and externally validated on the data from the other sites.

**Results:**

The performance of the algorithms, measured by the area under the curve of the internal validations as well as the corresponding external validations, ranged from 0.77 to 0.80.

**Conclusions:**

Machine learning models provide a robust and generalizable approach in automated risk signaling technology to identify cases at risk of poor treatment outcomes. The results of this study hold substantial implications for clinical practice by demonstrating that the performance of a model derived from one site is similar when applied to another site (ie, good external validation).

## Introduction

### Optimizing Health Care Systems

One of the main challenges in designing an efficient health care system is to prevent offering too many resources to some patients and too little to others. In other words, the challenge is to maximize the opportunity for appropriate care at an individual level [1]. The recent strive for precision or personalized medicine aims to improve health care systems by tailoring treatments to patients more effectively. Patients are grouped in terms of their expected treatment response using diagnostic tests or techniques [2]. However, precision medicine remains a challenge in mental health care because treatments are effective *on average,* but it is difficult to predict exactly whom they will work for [3,4]. Stepped care principles provide a framework to allocate limited health care resources and have been proven to be cost-effective for depression and anxiety [5,6]. In stepped care, treatments start with low intensity unless there is a reason to intensify. Such reasons are identified during treatment when there is a lack of confidence in a positive outcome given the current treatment trajectory. To this extent, routine outcome monitoring (ROM) could be used to observe patterns of early treatment response and identify which patients will probably not benefit from their current treatment [7,8].

### Identification of Nonresponders

The system can be improved by earlier and more accurate identification of those nonresponders so that patients do not have to endure periods of care in which they do not improve and could potentially lose interest and drop out. On top of that, scarce health care resources are not wasted by engaging in treatment without the desired effect. However, misclassification comes with a cost. Incorrectly classifying patients as needing more intensified treatment results in the unnecessary use of health care resources on patients who would have benefited from a shorter low-intensity treatment. In many Dutch clinics providing basic mental health care, ROM measurements are part of routine care. This raises the question of whether these ROM data could be used to provide accurate prognostic feedback and support a clinician in maximizing the opportunity for appropriate care on the individual level.

### Predicting Outcomes With Machine Learning During Treatment

Techniques from the field of machine learning are aimed at making accurate predictions based on patterns in data. Machine learning can help to identify robust, reproducible, and generalizable predictors of treatment response [3,9-11], and has already been used in health care research, for example, in predicting health care costs and outcomes [12-15]. By discovering associations and understanding patterns and trends within the data, machine learning has the potential to improve care. Machine learning permits a finer detection of which patients are at an elevated risk of experiencing persistent poor and costly health outcomes, and may thus give impetus to a more efficient, personalized, and proactive type of mental health care. Inspired by this knowledge, the study aims to use machine learning on ROM data as a feedback device to signal which patients have an elevated risk of a poor response to treatment [16]. However, the use of complex data, and the associated increasingly complex models, challenges researchers to ensure that these models are clinically interpretable rather than a “black box” [17,18].

### Independent Validation

After developing a prediction model, it is recommended to evaluate model performance in other clinical data that was not used to develop the model, as mentioned in the TRIPOD (Transparent Reporting of a Multivariable Prediction Model for Individual Prognosis or Diagnosis) statement. For example, such a validation would require researchers to have access to a similar data set (ie, in terms of predictor variables and outcomes) stemming from a similar population/clinic and compare model performance on this external independent data set (ie, cross-site design). The lack of independent validation is a major limitation of the extant machine learning literature in health care [19]. In a recent review on machine learning for suicide prediction, the majority of studies reviewed split the data into training and testing sets, whereas none of the studies used a cross-site design in which a model was trained using data from one site and evaluated using data from another [20]. Another recent review looking at applications of machine learning algorithms to predict therapeutic outcomes in depression concluded that most studies did not assess out-of-sample estimates of model fit, which limited their generalizability and likely overestimated predictive accuracy [15]. Therefore, the aim of this study was 2-fold: (1) to predict patients’ response to treatment in Dutch basic mental health care using limited commonly available data from routine care and (2) to compare the performance of these machine learning models across three different mental health care organizations in the Netherlands by using clinically interpretable models. By using commonly available data from routine care, the technical implementation of the model in clinical practice would be straightforward.

## Methods

### Study Design and Data Collection

Data on mental health treatment and outcomes were collected by a data collection protocol. Mental health care sites from 6 regions in the Netherlands were involved. Patients were treated for mild to severe mental health problems, low risk of suicide, or dangerous behavior. The data set consisted of patient records with a completed treatment from 2014 to 2018. A completed treatment in this setting consists of around 5-12 sessions [21]. The protocol consisted of a predefined set of variables with clear definitions and coding for each variable.

For treatment records to be included in this study, the availability of at least the ROM data as well as certain other variables that could be used for predictions was required. As ROM questionnaires are not mandatory in routine care, ROM data were not available for all patients at all measurements. Records were included when ROM data were available at the start of, during, and at the end of treatment. Of the 6 participating regions, 3 had sufficient treatment records (>1000) with nonmissing values and were included in the study (region 1: n=3020; region 2: n=1484; region 3: n=1948). In each region, patients were treated in multiple settings in both urban and rural areas. A set of 26,912 records had to be excluded from the three sites because there was a missing ROM measurement at either the start or end, such that the outcome could not be determined, or there was no measurement during treatment, such that early treatment response patterns could not be determined. To assess the comparability of the included and excluded treatment records in our analysis, a comparison was made regarding age, sex, diagnosis, and baseline severity between both groups (Table 1).

**Table 1.** Comparison of patient characteristics between the included and excluded treatment records.

		Included (n=6452)	Excluded (n=26,912)
**Sex, n (%)**			
Female	4077 (63.2)	16,872 (62.7)
	Male	2375 (36.8)	10,040 (37.3)
**Age category (years), n (%)**			
	<30	1978 (30.7)	8671 (32.2)
	30-40	1541 (23.9)	6701 (24.9)
	40-50	1238 (19.2)	5298 (19.7)
	50-60	1154 (17.9)	4119 (15.3)
	≥60	541 (8.4)	2123 (7.9)
**Diagnosis group, n (%)**			
	Anxiety	2588 (40.1)	9955 (37)
	Depression	2585 (40.1)	10,831 (40.2)
	Other	1279 (19.8)	6126 (22.8)
Total OQ-45.2^a^ score baseline, mean (SD)		80.36 (21.18)	80.60 (23.23)

a OQ-45.2: Outcome Questionnaire.

### Data Description

This study used treatment records, as opposed to patient records. A treatment record was started whenever a patient began treatment within one of the participating centers. As a result, some patients could have multiple treatment records (355/6452, 5.5% of the records were not unique). ROM assessed the development in symptom severity and functioning using the standardized Dutch version of the Outcome Questionnaire (OQ-45.2) [22]. The OQ-45.2 contains three subscales: Symptom Distress, Interpersonal Relations, and Social Role. The psychometric properties of the Dutch OQ-45.2 are adequate [23].

The idea of this study was to support a stepped care framework by predicting, during treatment, undesired outcomes at the end of treatment. These predictions can trigger a reconsideration of the chosen treatment plan to improve the probability of a desired outcome after finishing the treatment. Desired treatment outcomes are highly personal and dependent on the type of treatment and setting. For this study, we choose to define undesired outcomes as nonimprovement. Based on the principles of reliable change [24], we defined nonimprovement as improving less than a medium effect size on the Symptom Distress subscale of the OQ-45.2 [25]. Our study used data from the so-called *basic mental health care* in the Netherlands. Basic mental health care is cost-effective short-term mental health care with an average Cohen *d* effect size of 0.9 [21]. Despite this high effect size, the aim of this short-term treatment of 5-12 sessions is primarily to increase self-direction and get patients back on track without care as soon as possible. In this study, individual treatment goals were unknown, and therefore, it was decided to define nonimprovement as less than a medium effect size. This is a little more than half of the average improvement in this mental health care setting. Our clinical outcome was derived from the observed change in the Symptom Distress scale on the OQ-45.2. Patients with less than half of an SD improvement in symptom severity at the end of treatment were classified as having an “undesired clinical outcome” (called *nonimprovement* henceforth). With the SD of the Symptom Distress subscale in a Dutch clinical population being 16 [23], nonimprovement was defined as a patient not improving at least 8 points on the Symptom Distress subscale of the OQ-45.2.

An early change was defined as the difference in ROM at baseline and the first ROM during treatment. For both the summed scale scores on the OQ-45.2 as well as the individual items, early change variables were created. Besides the ROM data, a set of clinical and demographic variables were included for prediction such as main diagnosis, age, and living condition. The total set consisted of 163 variables, of which 144 were related to the scores on the OQ-45.2 and 19 to the context of the patient.

### Modeling and Validation Strategy

The data set was split across all included locations so that models could be trained on a single location and externally validated on each of the other locations. Nonimprovement was predicted for each location separately based on all available predictors using least absolute shrinkage and selection operator (LASSO) models. LASSO was used both to guarantee interpretability for intended model users and to facilitate explicit comparison between prediction models built in different locations. Moreover, as several measures were derived from the same questionnaire, this could have led to multicollinearity between predictors in the data set. LASSO is a technique that has been argued to be able to deal with multicollinearity and still provide stable and interpretable estimators [26]. All numeric variables were centered and scaled.

Using 10-fold cross-validation with 10 repeats, the optimal hyperparameter was determined by considering 100 possible penalty values (ie, λ) between 0.001 and 1000. For the LASSO with the optimized penalty, the probability threshold was tuned by optimizing *F*_1_-scores over 36 possible probability values between 0.3 and 0.65. The final LASSO model selected for each site was then applied to each of the other sites for model assessment, reporting sensitivity, specificity, positive predictive value (PPV), negative predictive value (NPV), and area under the curve (AUC) using the optimized probability threshold.

Bootstrapping was used to estimate model performance in the site in which the model was built to have an internally validated measure of model performance to compare with the two externally validated measures of model performance by estimating CIs for all performance scores (ie, sensitivity, specificity, PPV, and NPV). The bootstraps were performed by sampling each data set 1000 times with replacement, resulting in 1000 simulated data sets for each site. The final LASSO model of each of the 3 site-specific models was then applied to the bootstrapped data set, resulting in 1000 confusion matrixes per site. Next, the 2.5 percentile and 97.5 percentile for each performance indicator (ie, sensitivity, specificity, PPV, and NPV) were used to determine the 95% CI for each estimate.

All analyses were performed in R (version 4.0.0; R Foundation for Statistical Computing) [27]. The package *caret* was used to build the models [28]. The package *glmnet* was used to perform the LASSO regression [29]. The package *pROC* was used to analyze the AUCs [30].

### Ethical Considerations

Since the database was anonymized with statistical disclosure control techniques [31], there was no need for informed consent or approval by a medical ethics committee (Dutch Civil Law, Article 7:458).

## Results

The total data set used in the analyses contained information on 6452 treatment records and included anonymized demographic variables, care-related variables, and information about the severity and types of complaints. The characteristics of the patient populations within each site are shown in Table 2. There are notable differences between baseline symptom severity, the distribution of the main diagnosis, and the percentage of patients with a paid job between sites.

**Table 2.** Overview of research population (n=6452).

			Region 1 (n=3020)	Region 2 (n=1484)	Region 3 (n=1948)	*P* value
**Care-related variables**						
	Nonimprovement, n (%)		1028 (34.04)	499 (33.63)	577 (29.62)	.003
	Treatment duration (days), mean (SD)		145.19 (64.87)	208.00 (78.35)	205.78 (77.52)	<.001
	Treatment sessions (n), mean (SD)		9.73 (2.92)	13.15 (4.03)	11.21 (4.34)	<.001
**Type and severity of complaints**						
	Baseline symptom severity score, mean (SD)		51.42 (13.94)	52.16 (13.45)	48.72 (13.65)	<.001
	Baseline social role score, mean (SD)		13.76 (5.06)	14.37 (4.97)	13.79 (5.06)	<.001
	Baseline interpersonal relations score, mean (SD)		15.29 (6.08)	17.01 (6.50)	15.28 (6.11)	<.001
	Baseline total OQ-45^a^ score, mean (SD)		80.47 (21.25)	83.54 (20.76)	77.79 (21.07)	<.001
	**Diagnosis group, n (%)**					<.001
		Anxiety	1300 (43)	562 (37.9)	726 (37.3)	
		Depression	1142 (37.8)	568 (38.3)	875 (44.9)	
		Other	578 (19.1)	354 (23.9)	347 (17.8)	
**Demographic variables, n (%)**						
	**Sex**					.14
		Female	1878 (62.2)	934 (62.9)	1265 (64.9)	
		Male	1142 (37.8)	550 (37.1)	683 (35.1)	
	**Age category (years)**					<.001
		<30	954 (31.6)	505 (34)	519 (26.6)	
		30-40	694 (23)	369 (24.9)	478 (24.5)	
		40-50	577 (19.1)	249 (16.8)	412 (21.1)	
		50-60	556 (18.4)	241 (16.2)	357 (18.3)	
		≥60	239 (7.9)	120 (8.1)	182 (9.3)	
	**Origin**					<.001
		Native	2838 (94)	1 (0.1)	343 (17.6)	
		Immigrant	68 (2.3)	0 (0)	97 (5)	
		Unknown	114 (3.8)	1483 (99.9)	1508 (77.4)	
	**Marital status**					<.001
		Not married	1612 (53.4)	50 (3.4)	969 (49.7)	
		Married	1052 (34.8)	24 (1.6)	747 (38.3)	
		Divorced/widowed	356 (11.8)	8 (0.5)	224 (11.5)	
		Unknown	0 (0)	1402 (94.5)	8 (0.4)	
	**Living situation**					<.001
		Alone	981 (32.5)	35 (2.4)	571 (29.3)	
		With partner	1638 (54.2)	43 (2.9)	1100 (56.5)	
		Child	248 (8.2)	9 (0.6)	186 (9.5)	
		Other	151 (5)	6 (0.4)	83 (4.3)	
		Unknown	2 (0.1)	1391 (93.8)	8 (0.4)	
	**Paid job**					<.001
		Employed	1071 (35.5)	392 (26.4)	536 (27.5)	
		Not employed	1949 (64.5)	831 (56)	1412 (72.5)	
		Unknown	0 (0)	261 (17.6)	0 (0)	

a OQ-45.2: Outcome Questionnaire.

The nonzero LASSO coefficients are shown in Table 3. The most important coefficients, in terms of relative coefficient size, were related to early changes in the Symptom Distress subscale of the OQ-45.2, and the change in the total score of the OQ-45.2. The self-blame measurement at the start of treatment was the only other nonzero coefficient at each of the 3 regions. The coefficient for paid employment stands out in the region 1 model, and age had a notable coefficient in regions 1 and 3. Furthermore, the models contained smaller nonzero coefficients that varied between each site (eg, some OQ-45.2 variables were nonzero in some of the models but not in all of the models). The results of the hyperparameter tuning are shown in Table 4. As shown, the threshold to define a positive class was set between 0.30 (region 4) and 0.34 (region 3), with λ varying from 0.02 (region 5) to 0.16 (region 3).

**Table 3.** Nonzero least absolute shrinkage and selection operator coefficients of the three models.

		Region 1	Region 2	Region 3
Intercept		–0.59	–0.76	–1.14
Age		0.05	—^a^	0.04
Number of days between referral and first appointment (waiting queue)		—	0.05	—
Employment (paid job)		–0.39	–0.02	—
Nuisance on job (yes, very much)		—	–0.12	—
Work absence (unknown)		—	—	0.11
**OQ-45.2^b^ start measurement**				
	Self-blame	–0.08	–0.01	–0.07
	Feeling week	—	–0.01	—
	Happiness	—	0.05	—
	Disturbing thoughts	–0.11	—	—
	Stomach	—	—	–0.05
	Relationships	–0.01	—	—
	Sadness	–0.03	—	—
**OQ-45.2 middle measurement**				
	Suicidal thoughts	—	—	0.03
	Enjoyment	—	—	–0.01
	Relationships	–0.07	—	–0.01
**OQ-45.2 early change**				
	Stamina	—	—	0.01
	Satisfaction in work or school	–0.01	—	–0.05
	Disturbing thoughts	—	—	0.03
	Stomach	—	—	—
	Hearth	0.01	—	—
	Sleeping	0.03	—	—
	Sadness	0.03	—	—
	Relationships	—	–0.02	—
	Headaches	—	—	0.03
SD OQ-45.2 score (change)		0.97	0.81	1.09
Total OQ-45.2 score (change)		0.07	0.15	—

a Not applicable.

b OQ-45.2: Outcome Questionnaire.

**Table 4.** The parameter settings of the three models.

	Lambda	Probability
Model region 1	0.16	0.34
Model region 2	0.03	0.3
Model region 3	0.02	0.32

The performance of the three models is shown in Table 5. Each model (row) has been evaluated internally and two times externally. Each site (columns) has been used three times: one time for internal validation and two times for the external validation of the other models. The diagonal contains the three internal validations. The CIs of the AUCs overlap, which indicate that there were no significant differences in the overall performances of the models. The AUCs of the three models in the three internal validations were 0.77 (region 2) and 0.80 (regions 1 and 2). The AUCs of the six external validations ranged from 0.77 to 0.80. An overview of the associated confusion matrixes is attached in Multimedia Appendix 1.

**Table 5.** Comparison of internally (diagonal) and externally validated results within each site with 1000 bootstrapped CIs for regions 1, 2, and 3.

Metrics		Region 1 validation	Region 2 validation	Region 3 validation
**Region 1 model**				
	Sensitivity (95% CI)	0.784 (0.760-0.809)	0.762 (0.725-0.800)	0.780 (0.747-0.813)
	Specificity (95% CI)	0.698 (0.676-0.719)	0.647 (0.617-0.676)	0.673 (0.650-0.697)
	Positive predictive value (95% CI)	0.572 (0.545-0.600)	0.522 (0.486-0.560)	0.501 (0.471-0.534)
	Negative predictive value (95% CI)	0.862 (0.846-0.880)	0.843 (0.818-0.868)	0.879 (0.859-0.898)
	AUC^a^ (95% CI)	0.799 (0.783-0.816)	0.771 (0.746-0.794)	0.799 (0.778-0.819)
**Region 2 model**				
	Sensitivity (95% CI)	0.841 (0.818-0.863)	0.824 (0.789-0.856)	0.868 (0.844-0.896)
	Specificity (95% CI)	0.584 (0.563-0.606)	0.586 (0.554-0.615)	0.548 (0.520-0.574)
	Positive predictive value (95% CI)	0.511 (0.486-0.534)	0.502 (0.466-0.533)	0.447 (0.419-0.477)
	Negative predictive value (95% CI)	0.877 (0.860-0.893)	0.868 (0.841-0.892)	0.908 (0.890-0.927)
	AUC (95% CI)	0.782 (0.765-0.799)	0.774 (0.749-0.798)	0.792 (0.772-0.813)
**Region 3 model**				
	Sensitivity (95% CI)	0.696 (0.667-0.726)	0.673 (0.633-0.716)	0.742 (0.705-0.779)
	Specificity (95% CI)	0.749 (0.730-0.768)	0.726 (0.699-0.754)	0.732 (0.708-0.754)
	Positive predictive value (95% CI)	0.589 (0.561-0.617)	0.554 (0.517-0.596)	0.538 (0.503-0.573)
	Negative predictive value (95% CI)	0.827 (0.809-0.846)	0.814 (0.789-0.841)	0.871 (0.850-0.890)
	AUC (95% CI)	0.787 (0.771-0.803)	0.768 (0.744-0.792)	0.802 (0.782-0.822)

a AUC: area under the curve.

## Discussion

### Evaluation of Three Models at 3 Sites

The aim of this study was to use machine learning to predict which patients would not substantially benefit from treatment across 3 different mental health care organizations in the Netherlands by using clinically interpretable models. This study used a cross-site design in which the performance of a model developed in one site was compared to the model performance on an external independent data set (ie, 3 × 3 cross-site design, as per the TRIPOD statement). Data from ROM, among other clinical and demographic data, were used for the predictions.

Both the AUC of the internal validations of the three models and the corresponding external validations were in the range of 0.77 to 0.80, indicating fair to good model performance [32]. In addition, the CIs of the AUCs overlapped in each of the 9 evaluations, indicating that the performance estimates were robust and likely to be generalizable to different settings. This could be explained by the fact that LASSO regression is known to be less prone to overfitting compared to other machine learning algorithms, and when evaluated with 1000 times bootstrapping, the internal validations give a good indication of overall performance.

All three models generalized well to the other sites. This is an interesting finding and a promising result for the scalability of the implementation of machine learning models. Decentralized data can be gathered, within the boundaries of the General Data Protection Regulation. A model can be developed within the context of one site and then be exported to other sites, even if those other sites differ in certain characteristics. For example, in this research, the 3 sites differed in geographical location from more rural to urban. The patient populations differed, with some significant differences in the distribution of important variables such as main diagnosis, baseline symptom severity, and percentage of patients with paid employment. The data sources differed in the type of electronic health record system used in clinical practice. Despite these substantial differences, we were able to develop three robust machine learning models with acceptable AUCs that could be applied in all 3 settings.

The sensitivity and specificity of the three models were consistent in each of their external validations. There were differences in these metrics between models, mainly caused by a trade-off between sensitivity and specificity when evaluating model performance with metrics from the confusion matrix. The models of regions 1 and 2 were more shifted toward a higher sensitivity and the model of region 3 toward a higher specificity. However, these differences were a shift in the balance rather than an *absolute difference* between the models, as was indicated by the comparable AUCs.

To give some insight into the practical utility of the model, the results can be translated to a hypothetical clinical scenario. Imagine a health care professional with a caseload of 30 patients working in region 2, with a model created in region 1. About 10 of the 30 patients will not improve according to our data (34%). The model is used by the clinician to support the identification of potential nonimproving patients during treatment. With a sensitivity of 0.76 and a specificity of 0.65 (the results of model 1 applied to region 2), 15 patients will be classified as nonimprovers and 15 will be classified as improvers. Among the improvers, 13 of them will actually improve (ie, NPV=0.84), and among the nonimprovers, 8 of them would actually not improve (ie, PPV=0.52). For half of the patients who are classified as nonimprovers, therefore, the discussion would not be necessary at that time. So the question is whether these models are already good enough to actually use in practice. The idea is that when the model indicates that a patient is on track, there is little reason to change treatment. When the model indicates an elevated risk of nonimprovement, the clinician and patient should discuss the situation and adapt treatment plans if necessary. It is therefore important to see such machine learning models not as black-and-white decision tools but as complementary tools in the identification and stratification of patients in need of more or less care.

### Predictive Variables

Although this research was aimed at making predictions, rather than explaining relations, we used LASSO regression to inform clinicians about how the algorithm works. In the health care setting, this is important as health care professionals often want to understand which parameters affect and how they contribute to a prediction [33]. By looking at the coefficients of each LASSO model, it can be concluded that the algorithms rely on the variables’ early change in the Symptom Distress subscale and the total scores of the OQ-45.2, as well as having a paid job at the start of the treatment and age. In a paper by McMahon [34], several other studies are mentioned in which early symptom improvement, or lack of it, has been associated with psychiatric treatment outcomes. In a study by Lorenzo-Lucas et al [35], being unemployed, among other factors, predicted a lower likelihood of recovery. There were certain individual OQ-45.2 questionnaire items that were associated with nonzero LASSO coefficients. However, these items differed between the sites, and the size of the coefficients were relatively low. We are, therefore, reluctant to generalize findings on these individual OQ-45.2 items, with small nonzero coefficients, to future prediction research.

The high relative importance of the early change variable (ie, in terms of the absolute values of the coefficients) is likely to contribute to good external model validation, as it is a straightforwardly defined predictor that is less likely to be subject to sampling variation. Furthermore, given the high importance of early change in the model, one could even advocate for an alternative simpler predictive model (ie, a “rule of thumb”) using early change only (or combined with weaker predictors, eg, age and employment status).

### Strengths and Limitations

The main strength of this study is that we used a 3 × 3 cross-site design to develop and evaluate the algorithms, resulting in three models with an independent validation of their performance. In addition, LASSO regression was used, which is a parametric approach, resulting in a prediction model that is still relatively easy to interpret. Moreover, LASSO is less prone to overfitting, which increased the generalizability of the results. Furthermore, with the use of a data protocol with clear data definition descriptions, we could use readily available data from routine care in the Netherlands, meaning that our approach could easily be adopted in other Dutch basic mental health care organizations using ROM (the R scripts to build and validate the models are available on request). This study has a number of limitations that need to be acknowledged. First, we limited our analysis to treatment records with complete data only. In addition, we could not use every variable described in the data protocol because of missing values on these variables in one of the sites. Moreover, we had to exclude a large set of records because of missing data on the OQ-45.2. However, the excluded group of patients did not substantially differ in sex, age, diagnosis, or baseline symptom severity. Nonetheless, we would like to emphasize that our models cannot be directly applied to other patient populations. Second, our data did not contain information on whether the outcome of the ROM had already been used to alter the treatment strategy. This would underestimate the impact of early change, as patients with only minor or no clinical improvements would have been given a possibly more intensive treatment for them to respond to the treatment. Third, although it is difficult to estimate the required sample size for developing a prognostic model, our data had a relatively small sample size [36]. Fourth, this study chose to define an undesired outcome as improving with less than a medium effect size. However, the definition of an undesired outcome is subjective and will differ between different types of treatment settings. Therefore, our definition cannot directly be generalized to other settings, and each research should make an effort to define a relevant undesired outcome for that domain with experts from clinical practice.

This study was performed within the context of a stepped care framework, in which treatment optimization is required during treatment. Our models heavily rely on predictors derived from early change patterns and can, therefore, not be applied at the start of treatment. Other research could analyze which type of predictors are more suited for a matched care framework and to what extent accurate predictions can be made in treatment response.

### Conclusion

Machine learning models provide a robust and generalizable approach in automated risk signaling technology to identify cases at risk of poor treatment outcomes. The results of this study hold substantial implications for clinical practice by demonstrating that the performance of a model derived from one site is similar when applied to another site (ie, good external validation). This is a promising result for the scalability of machine learning models developed in single-center studies. Our findings confirm that routine monitoring provides valuable information that can be used in prognostic models to predict treatment outcomes. Such prognostic models can be used as complementary tools for practitioners in a stepped care framework.

## Supplementary material

10.2196/44322 Multimedia Appendix 1 Confusion matrix results.

## Abbreviations

AUC: area under the curve
LASSO: least absolute shrinkage and selection operator
NPV: negative predictive value
OQ-45.2: Outcome Questionnaire
PPV: positive predictive value
ROM: routine outcome monitoring
TRIPOD: Transparent Reporting of a Multivariable Prediction Model for Individual Prognosis or Diagnosis
