# Supplementary material for: Predicting Undesired Treatment Outcomes With Machine Learning in Mental Health Care: Multisite Study
Source: JMIR Med Inform. 2023 Aug 23;11:e44322. doi: 10.2196/44322 (PMC10466445; doi:10.2196/44322)
Supplement: Multimedia Appendix 1 [file medinform-v11-e44322-s001.docx]

**Appendix 1. Confusion matrix results**

|  |  | *actual* |  |
| --- | --- | --- | --- |
|  |  | non-improvement | improvement |
| *predicted* | non-improvement | 806 | 222 |
|  | improvement | 602 | 1390 |

**Table 1. Confusion matrix result model 1 site 1.**

|  |  | *actual* |  |
| --- | --- | --- | --- |
|  |  | non-improvement | improvement |
| *predicted* | non-improvement | 380 | 119 |
|  | improvement | 348 | 637 |

**Table 2. Confusion matrix result model 1 site 2.**

|  |  | *actual* |  |
| --- | --- | --- | --- |
|  |  | non-improvement | improvement |
| *predicted* | non-improvement | 450 | 127 |
|  | improvement | 448 | 923 |

**Table 3. Confusion matrix result model 1 site 3.**

|  |  | *actual* |  |
| --- | --- | --- | --- |
|  |  | non-improvement | improvement |
| *predicted* | non-improvement | 865 | 163 |
|  | improvement | 828 | 1164 |

**Table 4. Confusion matrix result model 2 site 1.**

|  |  | *actual* |  |
| --- | --- | --- | --- |
|  |  | non-improvement | improvement |
| *predicted* | non-improvement | 411 | 88 |
|  | improvement | 408 | 577 |

**Table 5. Confusion matrix result model 2 site 2.**

|  |  | *actual* |  |
| --- | --- | --- | --- |
|  |  | non-improvement | improvement |
| *predicted* | non-improvement | 501 | 76 |
|  | improvement | 620 | 751 |

**Table 6. Confusion matrix result model 2 site 3.**

|  |  | *actual* |  |
| --- | --- | --- | --- |
|  |  | non-improvement | improvement |
| *predicted* | non-improvement | 716 | 312 |
|  | improvement | 499 | 1493 |

**Table 7. Confusion matrix result model 3 site 1.**

|  |  | *actual* |  |
| --- | --- | --- | --- |
|  |  | non-improvement | improvement |
| *predicted* | non-improvement | 336 | 163 |
|  | improvement | 270 | 715 |

**Table 8. Confusion matrix result model 3 site 2.**

|  |  | *actual* |  |
| --- | --- | --- | --- |
|  |  | non-improvement | improvement |
| *predicted* | non-improvement | 428 | 149 |
|  | improvement | 368 | 1003 |

**Table 9. Confusion matrix result model 3 site 3.**
